# The Effects of Salts and Osmoprotectants on Enzyme Activities of Fructose-1,6-biphosphate Aldolases in a Halotolerant Cyanobacterium, *Halothece* sp. PCC 7418

**DOI:** 10.3390/life10030023

**Published:** 2020-03-09

**Authors:** Siripat Ngoennet, Masaki Honda, Tanutcha Patipong, Takashi Hibino, Rungaroon Waditee-Sirisattha, Hakuto Kageyama

**Affiliations:** 1 Department of Microbiology, Faculty of Science, Chulalongkorn University, Payathai Road, Patumwan, Bangkok 10330, Thailand; siripat.ngoennet@gmail.com (S.N.); tanutchapatipong@gmail.com (T.P.); 2 Department of Chemistry, Faculty of Science and Technology, Meijo University, 1-501 Shiogamaguchi, Tenpaku-ku, Nagoya, Aichi 468-8502, Japan; honda@meijo-u.ac.jp (M.H.); hibino@meijo-u.ac.jp (T.H.); 3 Graduate School of Environmental and Human Sciences, Meijo University, 1-501 Shiogamaguchi, Tenpaku-ku, Nagoya, Aichi 468-8502, Japan

**Keywords:** fructose-1,6-bisphosphate aldolase, salt stress, halotolerant cyanobacteria, glycine betaine

## Abstract

The halotolerant cyanobacterium, *Halothece* sp. PCC 7418, possesses two classes of fructose-1,6-bisphosphate aldolase (FBA): H2846 and H2847. Though class I (CI)-FBA H2846 is thought to be associated with salt tolerance, the regulatory mechanisms, molecular characteristics, and expression profiles between H2846 and class II (CII)-FBA H2847 have scarcely been investigated. Here, we show that the accumulation of the H2846 protein is highly responsive to both up- and down-shock with NaCl, whereas H2847 is constitutively expressed. The activity of CI- and CII-FBA in cyanobacterial extracts is correlated with the accumulation patterns of H2846 and H2847, respectively. In addition, it was found that these activities were inhibited by NaCl and KCl, with CII-FBA activity strikingly inhibited. It was also found that the CI-FBA activity of recombinant H2846 was hindered by salts and that this hindrance could be moderated by the addition of glycine betaine (GB), whereas no moderation occurred with other potential osmoprotectant molecules (proline, sucrose, and glycerol). In addition, a phylogenetic analysis showed that CI-FBAs with higher similarities to H2846 tended to be distributed among potential GB-synthesizing cyanobacteria. Taken together, our results provide insights into the independent evolution of the CI- and CII-FBA gene families, which show distinct expression profiles and functions following salt stress.

## 1. Introduction

**Figure 1:**
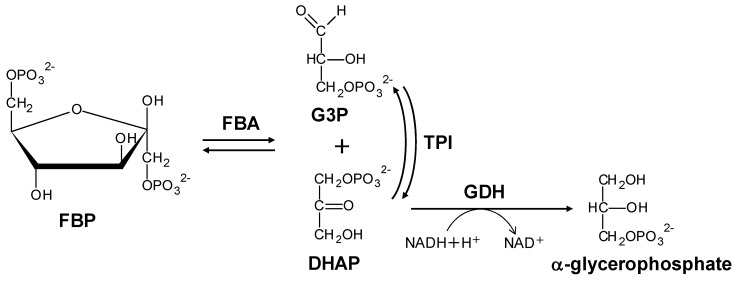
A coupled enzyme assay for the determination of FBP-cleavage activity. The enzymatic activity of FBA was measured by monitoring the decrease in the absorption of NADH at 340 nm. FBP, fructose 1,6-bisphosphate; FBA, fructose-1,6-bisphosphate aldolase; G3P, glyceraldehyde-3-phosphate; DHAP, dihydroxyacetone phosphate; TPI, triose phosphate isomerase; and GDH, glycerol phosphate dehydrogenase.

Fructose-1,6-bisphosphate aldolase (FBA), an enzyme, which is indispensable for glycolysis, gluconeogenesis, and the Calvin cycle, catalyzes the reversible aldol cleavage of fructose 1,6-bisphosphate (FBP) into glyceraldehyde 3-phosphate (G3P) and dihydroxyacetone phosphate (DHAP) (Figure 1) [1]. FBAs are classified into two distinct classes, according to their catalytic mechanism and independent evolutionary occurrence [2]. Class I (CI)-FBAs catalyze the reaction via the formation of a Schiff base, which attacks the C1 carbonyl group of G3P between the C2 carbonyl group of DHAP and the N6 amino group of an essential lysine residue in the active site. Class II (CII)-FBAs require divalent metal ions for a similar catalytic mechanism. It has been reported that CI-FBAs are commonly present in eukaryotic organisms, such as animals, plants, and green algae, but occur rarely in bacteria, whereas CII-FBAs are generally present in bacteria, archaea, and lower eukaryotes, such as fungi and some green algae [3].

Interestingly, based on genome sequence information, it has been shown that specific cyanobacterial species, including the cyanobacterium *Halothece* sp. PCC 7418 (*Halothece* 7418), possess CI-FBA in addition to CII-FBA [4]. Hereafter, we refer to *Halothece* 7418 CI-FBA and CII-FBA as H2846 and H2847, respectively, according to a previous report [4]. *Halothece* 7418, formerly identified as *Aphanothece halophytica* [5], is a halotolerant cyanobacterium, which was originally isolated from the Dead Sea, that can grow under high salinity concentrations of up to 3.0 M NaCl and an alkaline pH of 11 [6]. This strain is a remarkable example of cyanobacterial tolerance to high salt concentrations. To adjust their internal osmotic status under high salinity conditions, *Halothece* 7418 synthesizes and accumulates the osmoprotectant, glycine betaine (GB) via a three-step methylation reaction by two *N*-methyltransferases that utilizes glycine [7]. In addition to GB, it has been reported that another potential osmoprotectant, mycosporine-2-glycine (M2G), is also biosynthesized by *Halothece* 7418 [8,9]. However, the finding that the accumulated levels of GB were considerably higher than those of M2G [10] suggests that GB is the main osmoprotectant in *Halothece* 7418. Additionally, unique mechanisms for ion homeostasis in *Halothece* 7418 have been extensively investigated [11]. Very recently, we identified an H2846 protein as a salt-inducible CI-FBA in *Halothece* 7418 [4]. A phylogenetic analysis showed that H2846-like CI-FBAs are mostly present in cyanobacteria that inhabit hypersaline environments. The heterologous expression of H2846 but not H2847, a CII-FBA in *Halothece* 7418, could confer salinity tolerance to freshwater cyanobacterium. These results suggest a functional distinction between CI-FBA and CII-FBA in *Halothece* 7418, as well as the contribution of H2846 to salt tolerance mechanisms by the reinforcement of intracellular metabolic activities. However, the regulatory mechanisms, molecular characteristics, and expression patterns of these *Halothece* FBAs remain to be clarified. In the present study, we investigated the regulation of CI- and CII-FBA activity by confirming the levels of the accumulation of the H2846 and H2847 proteins in *Halothece* 7418 under salinity shock-treatment conditions. Our data revealed that only CI-FBA activity was highly responsive to both NaCl up- and down-shock treatments. The accumulation dynamics of CI-FBA (H2846) were highly induced upon salt stress. Furthermore, CI-FBA activity showed better resistance to salts than CII-FBA activity. Finally, we demonstrated that GB significantly alleviated the inhibitory effect of salt on the CI-FBA activity of the recombinant H2846 protein. These findings provide insights into the independent evolutionary history of the CI- and CII-FBA gene families, which exhibit distinct expression profiles and functions following salt stress. 

## 2. Materials and Methods 

### 2.1. Cyanobacterial Culture Conditions

The cyanobacterium *Halothece* 7418 was photoautotrophically grown under continuous illumination of 70 μE m^−2^ s^−1^ at 30 °C in a liquid BG11 plus Turk Island salt solution containing 0.5 M or 2.5 M NaCl. The medium was prepared according to a previously described recipe [12]. For the NaCl up-shock treatment, cells cultured in the media containing 0.5 M NaCl for at least 10 days were harvested by centrifugation and then resuspended in the media containing 2.5 M NaCl. Conversely, cells cultured in the media containing 2.5 M NaCl were moved into the media containing 0.5 M NaCl for the NaCl down-shock treatment.

### 2.2. Preparation of Rabbit Antiserum Directed Against H2847 Protein

A white rabbit was immunized four times with a total of 1.0 mg of synthesized peptide that corresponded to the region 289-306, REAAMKDPANFDPRHFLK, in the amino acid sequence of H2847 (Sigma-Aldrich Japan, Tokyo, Japan). The specificity of the antiserum for the H2847 protein was assessed by immunoblotting, which revealed that the antiserum specifically detected a band that was consistent with the predicted molecular mass (38.9 kDa) of H2847.

### 2.3. Preparation of Soluble Protein Extracts of Halothece 7418 for Measurement of FBA Activity

*Halothece* 7418 cells were collected from 50 mL of the culture during the log phase (OD_730_ = 0.6~0.9) and then stored at −80 °C until use. Cells were suspended in 700 μL of 50 mM Tris-HCl (pH 8.0) and sonicated on ice by using a VP-5s sonicator (TAITEC, Saitama, Japan) for a total of 40 s (repeated time-on/time-off of 10 s each time), with the output power set to 7. The samples were then centrifuged at 22,000 × g for 10 min at 4 °C. The supernatant solutions were used as total soluble protein extracts. Protein concentration was determined by using a TaKaRa Bradford Protein Assay Kit (Takara Bio Inc., Shiga, Japan).

### 2.4. Preparation of Purified Recombinant H2846 Protein

The recombinant H2846 protein was prepared as previously described [4]. Briefly, the H2846 protein was expressed in a soluble form in *Escherichia coli* BL21, which harbored the expression vector pColdI-H2846; the protein was then extracted and purified. The purification process consisted of two chromatographic steps: affinity purification that used an Ni-NTA-spin kit column (Qiagen, Hilden, Germany) and size-exclusion chromatographic purification that used a HiLoad 16/600 Superdex 200 pg column (GE Healthcare Life Sciences, Little Chalfont, United Kingdom).

### 2.5. Measurement of FBA Activity

The FBP-cleavage activity of FBA was measured by using a coupled enzyme assay. The reactions that were involved in the assay are illustrated in Figure 1.

To measure CI-FBA activity, an assay was performed according to a previously described method [4]. Briefly, the reaction mixture contained 50 mM Tris-HCl (pH 8.0), 0.2 mM NADH, 1 unit of glycerol phosphate dehydrogenase and triose phosphate isomerase (GDH-TPI coupling enzyme; Sigma-Aldrich, St. Louis, MO, USA), 100 μM FBP, and 5 mM ethylenediaminetetraacetic acid (EDTA). EDTA was added to inhibit the CII-FBA activity. Following the addition of the *Halothece* 7418 soluble protein extract (20 μg of total protein) or the purified H2846 protein (1 μg) to the reaction mixture, a decrease in absorbance at 340 nm was monitored by using a UV-1800 spectrophotometer (Shimadzu, Kyoto, Japan). The total volume of the reaction mixture was 500 μL. The reaction was performed at 30 °C. 

To measure CII-FBA activity, a similar assay was carried out. However, the reaction mixture contained 2 mM MnCl_2_ to activate CII-FBA. Additionally, 10 mM sodium borohydride was supplied, rather than EDTA, to inhibit CI-FBA activity.

To investigate the effect of salts (NaCl or KCl) or osmoprotectants (GB, proline, sucrose, or glycerol) on FBA activity, these compounds were dissolved in the reaction mixture at the indicated concentrations before the enzyme solution was added. Prior to conducting the analysis for the effect of salt, it was confirmed that NaCl and KCl did not inhibit the activity of the GDH-TPI coupling enzyme by using G3P as a substrate (data not shown).

One unit of FBA activity was defined as the amount of FBA that was required for the cleavage of 1 μmol of FBP (equivalent to 2 μmol of oxidized NADH) in 1 min. The molar extinction coefficient for NADH (6220 M^−1^ cm^−1^) was used for the calculation.

### 2.6. Gel Filtration Chromatography of Halothece Extracts

*Halothece* 7418 cells that were cultured under 2.5 M NaCl conditions were collected from 130 mL of the culture during the log phase (OD_730_ = 0.6~0.9) and then stored at −80 °C until use. Cells were suspended in 2.4 mL buffer A (50 mM Tris-HCl (pH 8.0), 150 mM NaCl, 5 mM MgCl_2_) and sonicated on ice by using a VP-5s sonicator (TAITEC) for a total of 40 s (repeated time-on/time-off of 10 s each time), with the output power set to 7. The samples were then centrifuged at 22,000 × g for 15 min at 4 °C. The supernatant solutions were passed through a membrane filter (pore size: 0.45 μm) and then subjected to size-exclusion separation by using a HiLoad 16/600 Superdex 200 pg column. Buffer A was used as the eluent. Note that MgCl_2_ was used instead of MnCl_2_ in buffer A because manganese can be oxidized by Tris to form precipitate over a period of hours [13]. All protein chromatographic steps were performed by using the AKTA start system (GE Healthcare Life Sciences) at 4 °C. During separation, buffer A was applied at a flow rate of 2.0 mL/min, and separated protein fractions were continuously collected every 2.0 mL. The protein standards that were used for calibration were ferritin (440 kDa), aldolase (158 kDa), conalbumin (75 kDa), ovalbumin (43 kDa), chymotrypsinogen A (25 kDa), and ribonuclease A (13.7 kDa); all were purchased from GE Healthcare Life Sciences. The proteins in 1.0 mL aliquots of the fractions were precipitated with trichloroacetic acid (TCA), and then the precipitates were dissolved in a 50 μL SDS sample buffer and boiled for 5 min.

### 2.7. Immunoblotting Analysis

For the immunoblotting analysis of the *Halothece* 7418 soluble protein extracts, 3.0 μg of total protein samples were subjected to SDS-PAGE on 12% gels and blotted onto polyvinylidene difluoride (PVDF) membranes. For the immunoblotting analysis of gel filtration chromatographic fractions, 5 μL of each sample was used. H2846 and H2847 were analyzed with specific antisera at a 1:1000 dilution and detected by a colorimetric method by using the alkaline phosphatase-conjugated secondary antibody. Anti-H2846 antiserum was prepared previously [4].

### 2.8. Phylogenetic Analysis

Cyanobacterial H2846-like proteins were extracted by using the National Center for Biotechnology Information (NCBI) protein BLAST algorithm, by searching with the amino acid sequence of H2846 as a protein query. The reliability of the phylogenetic tree (Figure 7) was assessed with a bootstrap analysis with 1000 replicates.

## 3. Results and discussion

### 3.1. Protein Accumulation Dynamics of H2846 and H2847 Following Salinity Shock Treatment

**Figure 2:**
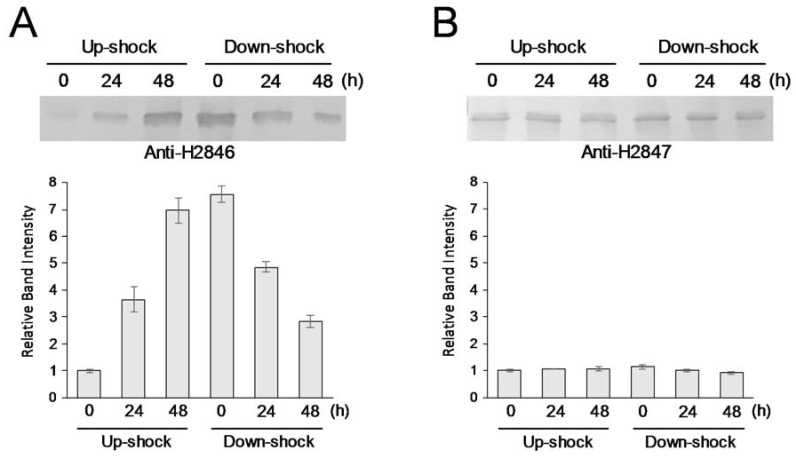
Accumulation dynamics of H2846 (**A**) and H2947 (**B**). Upper panels: Target FBAs were analyzed by immunoblotting by using corresponding antisera. Lower panels: densitometric data of the upper panels are shown. Each value represents the average of three independent analyses. Means ± SEM are shown.

First, we investigated the accumulation pattern of CI-FBA H2846 under salt-shock conditions in *Halothece* 7418 cells. In this study, both NaCl up-shock (0.5 M to 2.5 M) and down-shock (2.5 M to 0.5 M) treatments were tested. As shown in Figure 2A, the immunoblotting analysis revealed that H2846 accumulation was significantly enhanced following an up-shock, as previously reported [4], and the level increased by approximately seven-times within 48 h following treatment. By contrast, the salt down-shock treatment caused a rapid decrease in H2846 accumulation. Thus, H2846 accumulation was highly responsive to the salinity shock treatment.

The accumulation of CII-FBA H2847 was examined in parallel. To do this, antiserum against the H2847 protein was newly prepared for the immunoblotting analysis (see Materials and Methods). As shown in Figure 2B, although our previous data demonstrated a slight upregulation of *H2847* gene expression following NaCl up-shock treatment [4], the level of accumulation of the H2847 protein was unchanged under both the up- and down-shock-treated conditions. A constant amount of H2847 might function as housekeeping FBA, which is involved in fundamental metabolism in *Halothece* 7418. The fact that H2847-type CII-FBA is highly conserved across a broad range of cyanobacterial species [4] supports this hypothesis.

**Figure 3:**
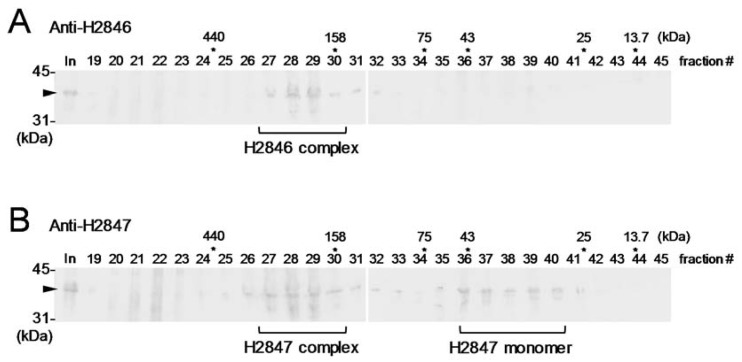
Complex formation of H2846 (**A**) and H2847 (**B**) in *Halothece* 7418 cells. The size-fractionated soluble protein extracts of *Halothece* 7418 by gel filtration analyses were subjected to immunoblotting by using corresponding antisera. The numbers upper the pictures indicate the number of fractions. The asterisks indicate the peaks of standard proteins and their native molecular size. ‘In’ indicates input, which was 3.0 mg of the total protein sample of the *Halothece* soluble protein extract that was prepared from the cells that were sampled 48 h following NaCl up-shock treatment. The arrowheads indicate the bands of target FBAs. The peak areas corresponding to putative monomeric form and/or complexes are marked under the bands.

In addition, we examined the complex formation of FBAs in *Halothece* cells that were cultured under salt stress (2.5 M NaCl) conditions. The gel filtration analysis followed by immunoblotting demonstrated that H2846 eluted as a single peak at a size larger than 158 kDa, as previously reported (Figure 3A) [4]. Through the use of HPLC-based size-exclusion chromatography, it has been suggested that H2846 forms a homohexamer by using a recombinant protein [4]. On the other hand, here, H2847 eluted as two peaks: one peak at a size larger than 158 kDa and another peak at a size between 25 and 43 kDa (Figure 3B). The predicted molecular mass of H2847 was 38.9 kDa, so it is likely that H2847 exists as both protein complexes and monomeric forms in almost the same ratio (Figure 3B). If the complex formed was a homo-oligomer, it should have been larger than a tetramer (155.6 kDa). This observation is interesting, because CII-FBAs of both bacterial and eukaryotic origin have been reported to commonly form homodimers [14]. 

### 3.2. FBA Activity under Salinity-Shocked Conditions in Halothece 7418

Next, the FBA activity of *Halothece* 7418 extracts was examined. To measure the activity of CI-FBA and CII-FBA, EDTA, and sodium borohydride were added as respective inhibitors of the opposite activity (see Materials and Methods).

**Figure 4:**
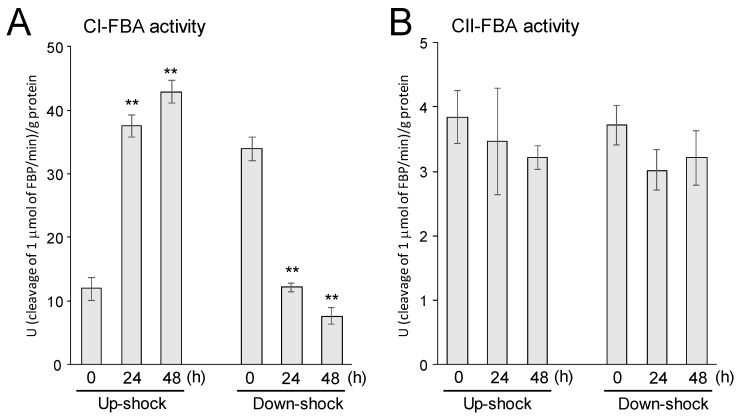
Class I FBA (CI-FBA) (**A**) and Class II FBA (CII-FBA) (**B**) activity of the *Halothece* extracts that were prepared from the cells that received salinity shock treatments. Each value represents an average of three independent analyses. Means ± SEM are shown. ** indicates that differences between changes in activity compared with time 0 were significant (*t*-test, *p* < 0.01). Note that neither NaCl nor KCl was mixed additionally in the reaction mixtures when the activities were measured.

As shown in Figure 4A, CI-FBA activity was upregulated and downregulated by up- and down-shock with NaCl, respectively. The patterns correlated well with the accumulation profile of the H2846 protein (Figure 2A).

To measure CII-FBA activity, divalent metal ion dependency was investigated first, because this class of FBAs requires divalent ions for their activity. For example, it was reported that cobalt (II) ions could have the best effect for activating CII-FBA, the sll0018 protein, in the cyanobacterium *Synechocystis* sp. PCC6803 [15]. To this end, eight divalent ions were tested in the presence of sodium borohydride, and it was found that the addition of manganese (II) ions resulted in the highest CII-FBA (H2847) activity in the *Halothece* 7418 extract (Table 1). The difference in divalent ion dependency between H2847 and sll0018 is an interesting subject that should be further clarified, since the amino acid sequences of these CII-FBAs were highly conserved (85% identity). Then, manganese (II) ions were added to the reaction mixtures for the CII-FBA assay by using the *Halothece* 7418 extracts. As a result, CII-FBA activity was not significantly changed by either the up- or down-shock treatments (Figure 4B). This result was in accordance with the accumulation pattern of the H2847 protein (Figure 2B).

### 3.3. NaCl- and KCl-inhibited FBA Activity of Halothece 7418 Extracts

**Figure 5:**
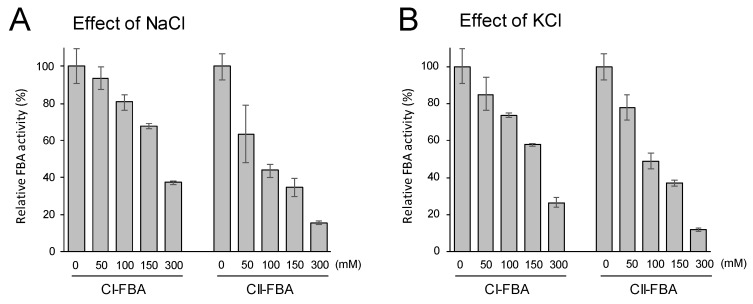
Effects of NaCl (**A**) and KCl (**B**) on the activity of FBA in the *Halothece* 7418 extracts. Relative values of CI- and CII-FBA activities are shown. Each value represents an average of three independent analyses. Means ± SEM are shown. The values at time zero of each dataset were set to 100.

*Halothece* 7418 can adapt to the surrounding salt concentration by regulating GB accumulation and unique mechanisms for ion homeostasis in the long term. The intracellular ion content in *Halothece* 7418 cells that were subjected to salt stress was experimentally determined. Previous reports have revealed low concentrations of intracellular ion contents, although there have been divergences [16]. For instance, Miller et al. reported that the intracellular sodium ion concentration was just 0.027 mM in *Halothece* 7418 cells that were cultured under 3000 mM NaCl conditions [17]. Reed et al. demonstrated that sodium and potassium ions were detected at 80–180 mM and 180–250 mM, respectively, in *Halothece* 7418 cells that were cultured under 156–934 mM NaCl conditions [18]. Incharoensakdi and Takabe reported that the intracellular chloride ion concentration in *Halothece* 7418 ranged from 35 to 150 mM when the NaCl concentration in the medium was changed from 0.5 to 2.0 M [19]. Thus, the salt concentration of the culture medium can affect the intracellular ion content in *Halothece* 7418. In addition, when the cells are exposed to a sudden salt shock, the intracellular salt accumulation can be transiently changed to achieve a rapid osmotic balance [16]. Intracellular salt might be problematic from an enzymatic point of view; for example, ribulose 1,5-bisphosphate carboxylase-oxygenase (RuBisCO) activity in the crude extracts that were prepared from *Halothece* 7418 was inhibited by approximately 80% in the presence of a 300 mM concentration of NaCl or KCl [20]. Likewise, the intracellular activities of H2846 and H2847 could be affected by these salts. Here, we investigated the effects of NaCl and KCl on the activity of FBA in extracts from *Halothece* 7418 (Figure 5). In these experiments, we tested salt concentrations of up to 300 mM. As shown in Figure 5A, NaCl inhibited both CI- and CII-FBA activity in a concentration-dependent fashion. Intriguingly, the resistance of CI-FBA activity to NaCl was stronger than that of CII-FBA activity. For example, CII-FBA activity was inhibited by 56%, whereas CI-FBA activity was inhibited by just 19% in the presence of 100 mM NaCl. A similar tendency was observed when using KCl (Figure 5B). In the presence of 300 mM KCl, CII-FBA activity was reduced to just 12%, but CI-FBA retained 27% of activity. These observations suggest that CI-FBA H2846 contributes more than CII-FBA H2847 to maintain basic metabolic activities in *Halothece* 7418 cells under salinity stress conditions. In other words, highly expressed CI-FBA H2846, which has resistance to salts, might compensate for intracellular FBA activity when the constantly expressed housekeeping CII-FBA H2847 is inhibited by salts under salinity stress condition. 

### 3.4. GB Alleviated the Inhibitory Effect of Salts on the CI-FBA Activity of Recombinant H2846 Protein

**Figure 6:**
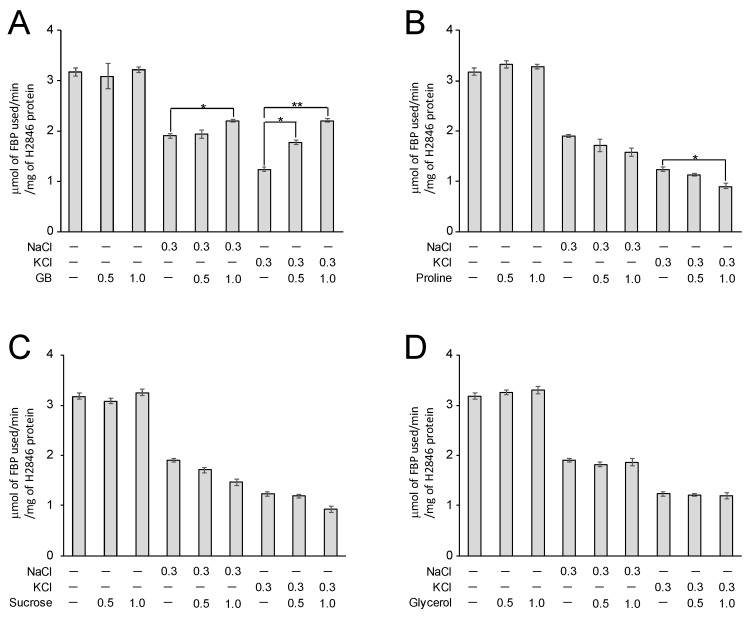
Effects of salts and osmoprotectant molecules on the CI-FBA activity of H2846. A recombinant H2846 protein was used for the activity measurements. The osmoprotectant molecules used were GB (**A**), proline (**B**), sucrose (**C**), and glycerol (**D**). The final concentration (M) of the added salts and osmoprotectants is indicated. Each value represents the average of three independent analyses. Means ± SEM are shown. * and ** indicate significant differences between changes in activity (*t*-test, *p* < 0.05 and *p* < 0.01, respectively).

Osmoprotectant molecules are important stabilizing agents for macromolecules, including proteins, in cyanobacteria [21]. *Halothece* 7418 biosynthesizes and accumulates GB in response to salinity stress. In addition to having a strictly osmotic function, GB enhances enzymatic activities in vitro [7] and has been demonstrated to have protective effects against various types of stresses [7,22]. It is worth testing whether GB has any effect on the enzymatic activity of salt-responsive FBAs. We therefore analyzed the effects of GB on CI-FBA activity by using a purified recombinant H2846 protein. According to previous reports [18,23], the intracellular concentration of GB can be up to 1000 mM in *Halothece* 7418. Therefore, we added GB to the reaction mixture at concentrations of 500 and 1000 mM. As shown in Figure 6A, the addition of GB alone did not affect the CI-FBA activity of H2846; however, in the presence of 300 mM NaCl or KCl, GB could have reduced the inhibitory effects of both NaCl and KCl on CI-FBA activity. In the presence of KCl, in particular, 1000 mM GB enhanced KCl-inhibited activity by 1.8 times (from 1.2 to 2.2 μmol of FBP used/min/mg of protein). Thus, GB might protect H2846 from potassium ions, since these ions seem to be more abundant than sodium ions in *Halothece* 7418 cells under high-salinity culture conditions [17,18,24]. Though detailed investigation of the protection mechanism of GB awaits further study, the zwitterionic property of GB could contribute to the stabilization of H2846 by exclusive interaction with its charged groups. Similarly, it has been reported that GB restores the potassium ion-induced loss of activity of glucose-6-phosphate dehydrogenase in *Halothece* 7418 [16]. GB was also shown to protect against KCl-induced damage to RuBisCO activity in *Halothece* 7418 [23].

Given the wide range of chemical properties of osmoprotectants, we employed three potential osmoprotectant molecules (proline, sucrose, and glycerol) to compare their effects with GB. Proline, which is a proteinogenic amino acid, is known to have an osmoprotective function [25]. It was found that the proline content increased in response to salinity concentration in the cyanobacterium *Anabaena variabilis* [26]. The disaccharide sucrose, as well as trehalose, is known to accumulate as a major compatible solute in freshwater cyanobacterial strains with low halotolerance [27]. Glycerol is well-known as an osmoprotectant that is naturally synthesized in yeast [28]. It has been reported that the cyanobacterium *Synechocystis* sp. PCC 6803 is also capable of bioproducing glycerol under mild salinity-stress conditions [29]. The most striking observation from using these four osmoprotectants was that only GB showed protective effects for FBA against NaCl and KCl stresses. As shown in Figure 6B–D, the addition of each osmoprotectant alone, at up to 1000 mM, did not show any significant effect on the CI-FBA activity of H2846. Likewise, in the case when both osmoprotectant and salt were added, none of the potential osmoprotectant molecules reduced the inhibitory effects of the salts. Note that only in the case where 1000 mM proline was added with KCl did the CI-FBA activity decrease compared with the condition in the presence of KCl alone. It is interesting that only GB could protect H2846 from salts. As described above, the zwitterionic characteristic of GB could affect this phenomenon; however, proline could also exist as zwitterion at physiological pH values. The structural differences, especially in positively charged parts, between GB and proline might be a reason for different protection effects.

Thus far, we have not succeeded in preparing recombinant *Halothece* CII-FBA H2847, as previously mentioned [4]. The enzymatic features of H2847, including effects of GB on its activity, are also interesting; therefore, this area should be examined in the future.

### 3.5. Relationship between the Distribution of H2846-type CI-FBA and the Ability to Synthesize GB in Cyanobacteria

**Figure 7:**
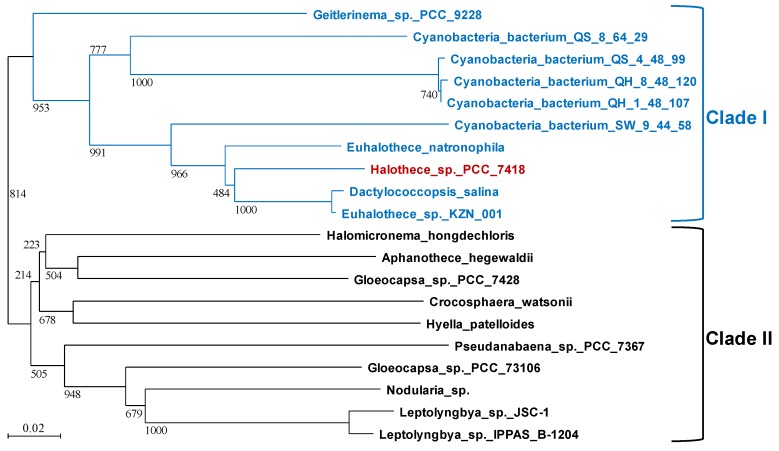
Distribution of the top 20 cyanobacterial strains that possess H2846-type CI-FBA proteins. The tree was generated by the neighbor-joining method by using ClustalW. Strain names in blue indicate cyanobacterial strains in clade I. Note that only *Halothece* 7418 in clade I is indicated by the color red. Strains in clade II are indicated in black. The bars represent evolutionary distance. The scale bar comprises 0.02 expected changes per amino acid site (0.02 substitutions/site).

According to the above results, GB could specifically relieve salt damage to CI-FBA H2846, at least among the potential osmoprotectant molecules that were tested. Figure 7 shows a phylogenetic tree of cyanobacterial H2846-like proteins. To depict the tree, we used the top 20 proteins that were hit by the NCBI protein BLAST algorithm, searching with the amino acid sequence of H2846 as a query. These proteins could be divided into two clades: clades I and II (Figure 7). *Halothece* 7418 was in clade I, along with cyanobacteria found in hypersaline environments, such as *Euhalothece* sp. and *Dactylococcopsis salina*. To explore the relationship between these clades and their ability to synthesize GB, we investigated the distribution of the GB-synthetic enzymes, glycine/sarcosine *N*-methyltransferase (GSMT) and dimethylglycine *N*-methyltransferase (DMT), among the cyanobacteria we analyzed. GSMT and DMT in *Halothece* 7418 were used as queries for the search. In this investigation, we also noted whether these two methyl transferases were neighboring to each other or not in order to judge the activities of their translation products for GB synthesis because it is known that corresponding genes of GSMT and DMT are adjacent to each other and form clusters in the genome of *Halothece* 7418, which is one of the best known GB-synthesizing cyanobacterial strains [30]. The results are summarized in Table 2. All species in clade I possessed GSMT- and DMT-like proteins, with similarities of more than 50% with *Halothece* GSMT and DMT. This suggested that the strains in clade I synthesized and accumulated GB, except for Cyanobacteria bacterium QS_8_64_29, in which two methyltransferase genes were not neighboring. On the other hand, it seemed that most of the strains in clade II did not possess a complete set of GB synthetic methyltransferases, although confirmation by experiment will be necessary to check that this is indeed the case. It should be noted that GSMT- and DMT-like proteins might be active for GB synthesis in *Gloeocapsa* sp. PCC 7428 in clade II, because these genes were neighboring in the genome as *Halothece* 7418, although the homologies were low (Table 2). 

## 4. Conclusions

In conclusion, we found interesting enzymatic properties of the CI-FBA H2846 protein in a halotolerant cyanobacterium, *Halothece* 7418. It was shown that the accumulation of the H2846 protein was highly sensitive to both NaCl up- and down-shock treatments, but it was also found that CII-FBA H2847 remained constant. Corresponding CI-FBA activity in *Halothece* 7418 extracts also fluctuated sharply in a salinity-dependent manner. In addition, FBA activity in the extracts was significantly inhibited by NaCl and KCl. Furthermore, the inhibitory effects of salts on the CI-FBA activity of recombinant H2846 protein were attenuated by the osmoprotectant molecule GB. Finally, it was demonstrated that cyanobacterial CI-FBAs with higher similarities to H2846 tended to be distributed among potential GB-synthesizing cyanobacterial strains. Therefore, H2846-type CI-FBAs are thought to act as metabolism-reinforcing proteins that could be protected by GB, in halotolerant cyanobacteria under high-salinity conditions.

## Figures and Tables

**Table 1:** Influence of divalent metal ions on FBA activity in the *Halothece* 7418 extract in the presence of 10 mM sodium borohydride (NaBH_4_).

Addition			Relative Activity (%) ^1^
Metal Ion ^2^	NaBH_4_^3^	EDTA ^4^	
-	-	-	298 ± 9.34 ^5^
-	+	-	ND ^6^
Mg^2+^	+	-	73.2 ± 4.88
Ca^2+^	+	-	68.3 ± 5.63
Mn^2+^	+	-	100 ± 10.8
Fe^2+^	+	-	ND ^6^
Co^2+^	+	-	ND ^6^
Cu^2+^	+	-	46.3 ± 2.44
Zn^2+^	+	-	ND ^6^
Mn^2+^	+	+	ND ^6^

^1^ The value obtained when using Mn^2+^ in the absence of EDTA was set to 100. Data are presented as mean values ± SD and were calculated from three sets of independent experiments. ^2^ Chloride salts of each indicated metal ion were added to a final concentration of 1 mM. ^3^ NaBH_4_ was added to a final concentration of 10 mM. ^4^ EDTA was added to a final concentration of 5 mM. ^5^ This activity shows CI-FBA activity in the *Halothece* extract. ^6^ Not detected.

**Table 2:** Summary of H2846-, glycine/sarcosine *N*-methyltransferase (GSMT)-, and dimethylglycine *N*-methyltransferase (DMT)-like proteins in the cyanobacterial strains that were used to generate Figure 7.

Clade	Cyanobacterial Strain Name	H2846-Like Protein		GSMT-Like Protein		DMT-Like Protein		Two MT Genes Are Neighboring
		Accession	Identity (%)	Accession	Identity (%)	Accession	Identity (%)	
I	Halothece sp. PCC 7418	WP_015226850.1	100.0	WP_015227493.1	100.0	BAC56940.1	100.0	yes
I	Euhalothece sp. KZN 001	PNW51873.1	91.35	PNW34186.1	92.83	PNW34185.1	91.70	yes
I	Dactylococcopsis salina	WP_015230391.1	90.78	WP_015231090.1	93.58	WP_015231089.1	91.70	yes
I	Euhalothece natronophila	WP_146295003.1	89.91	WP_146296755.1	89.06	WP_146296756.1	94.22	yes
I	Cyanobacteria bacterium SW_9_44_58	PSO48123.1	82.71	PSO48946.1	82.71	PSO48947.1	90.60	yes
I	Cyanobacteria bacterium QS_4_48_99	PSO81898.1	78.10	PSO78094.1	80.84	PSO78093.1	53.09	yes
I	Cyanobacteria bacterium QH_1_48_107	PSO51072.1	78.10	PSO56755.1	80.84	PSO56754.1	53.09	yes
I	Cyanobacteria bacterium QH_8_48_120	PSO71131.1	77.81	PSO78323.1	80.84	PSO78322.1	53.09	yes
I	Geitlerinema sp. PCC 9228	WP_071515292.1	76.68	WP_071515313.1	71.76	WP_071515314.1	57.45	yes
I	Cyanobacteria bacterium QS_8_64_29	PSP19570.1	76.16	PSP18883.1	79.54	PSP18856.1	57.45	no
II	Gloeocapsa sp. PCC 7428	WP_015191192.1	73.70	WP_015188788.1	32.43	WP_015190787.1	50.00	yes
II	Halomicronema hongdechloris	WP_080808545.1	73.12	WP_080807129.1	69.88	WP_080807129.1	52.54	no
II	Aphanothece hegewaldii	WP_106456509.1	72.25	N.H. ^1^	-	WP_106456849.1	39.39	no
II	Leptolyngbya sp. IPPAS B-1204	RNJ65497.1	71.97	RNJ64902.1	53.12	RNJ68485.1	35.20	no
II	Gloeocapsa sp. PCC 73106	WP_006527803.1	71.80	WP_006530277.1	26.67	WP_006530803.1	31.63	no
II	Pseudanabaena sp. PCC 7367	WP_015164428.1	72.33	N.H. ^1^	-	WP_015165920.1	38.18	no
II	Crocosphaera watsonii	WP_007311889.1	71.88	N.H. ^1^	-	WP_048316702.1	36.96	no
II	Leptolyngbya sp. JSC-1	WP_035999928.1	71.39	WP_051925143.1	53.12	WP_036010558.1	35.20	no
II	Nodularia sp.	TVP63635.1	70.93	TVP63914.1	29.63	TVP61242.1	36.36	no
II	Hyella patelloides	WP_144874142.1	70.52	WP_144866681.1	20.16	WP_144867980.1	33.71	no

^1^ N.H., not hi.
